# Can we eradicate Cysticercosis?

**Published:** 2011-01-10

**Authors:** Thierry Franchard, Remy Guebey, Julien Razafimahefa, Marcellin Andriantseheno, Harentsoaniaina Rasamoelina, Ronan Jambou

**Affiliations:** 1 Institut de la Francophonie pour la Médecine Tropicale, Vientiane, Lao PDR; 2 Institut Pasteur de Madagascar, Antananarivo, Madagascar; 3 Hôpital Befelatanana, Antananarivo, Madagascar; 4 Direction de la recherche zootechnique et vétérinaire, Antananarivo, Madagascar; 5 Département de Parasitologie Mycologie, Institut Pasteur, Paris, France

## 

Man is the only known definitive host of the tapeworm *Taenia solium* and becomes a carrier by eating undercooked pork contaminated with “Cysticercus cellulosae” (cysticerci). Pigs act as intermediate host and acquire cysticercosis by ingestion of eggs or proglottids from human feces, which develop into cysticerci within tissue mostly without causing clinical symptoms in the host. Cysticercosis occurs in man in a context of “Fecal peril” by ingestion of egg-contaminated soil, water or vegetation or by auto-infestation. In theory separation of swine from humans, good cooking practice and hygiene should lead straightforwardly to the eradication of the disease! However cysticercosis is still a major public health problem in endemic regions with more than 50 million infected people and is now a re-emerging disease in industrialized countries due to human migration. It is also the second cause of seizure in tropical countries. So what are the pitfalls in cysticercosis control and what can we do?

Cysticercosis affects free roaming pigs with access to sites contaminated with human feces. Development of good rearing practice guides will be of major impact. Only few tools are available for ante-mortem diagnosis of porcine cysticercosis and tongue palpation remains the most commonly used tool. Therefore, the development of a rapid diagnostic test, usable in villages, to test cattle will be the second weapon. However, this will need recombinant antigens. Diagnostic obstacles also affect human patients presenting with seizures. Scans and biological tests are not readily available leading to the repeated treatment of patients.

New target proteins are thus needed to develop these tests. With the sequencing of *T. solium* genome which will allow identification and production of recombinant protein a new step in the right direction was made. Now a large advocacy to raise funds in order to get this strategy on track is needed. Here we summarize the current state of the disease, practical issues linked to the organization of a feasible control system in developing countries and new data available all over the world and in particular in Madagascar to sustain this advocacy.

